# A Systematic Study on Digital Light Processing 3D Printing of 0-3 Ceramic Composites for Piezoelectric Metastructures

**DOI:** 10.34133/research.0595

**Published:** 2025-02-21

**Authors:** Huiru Wang, Qingbo Lai, Dingcong Zhang, Xin Li, Jiayi Hu, Hongyan Yuan

**Affiliations:** Department of Mechanics and Aerospace Engineering, Southern University of Science and Technology, Shenzhen 518055, China.

## Abstract

Digital light processing (DLP) is a high-speed, high-precision 3-dimensional (3D) printing technique gaining traction in the fabrication of ceramic composites. However, when printing 0-3 composites containing lead zirconate titanate (PZT) particles, a widely used piezoelectric ceramic, severe density and refractive index mismatches between the 2 phases pose challenges for ink synthesis and the printing process. Here, we systematically and quantitatively optimized DLP printing of PZT composites, streamlining process development and providing a solid theoretical and experimental foundation for broader applications of DLP technology. PZT particles were pretreated with air plasma to improve slurry uniformity and enhance stress transfer at the composite interface, leading to improved chemical modification, mechanical strength, and piezoelectric properties. We investigated the effects of key process parameters on printability and accuracy by analyzing the curing behavior of PZT–polymer composites. A quantitative model of the DLP curing process was introduced. Unlike stereolithography (SLA), DLP curing depth was found to depend on energy dose and light intensity, with higher intensities proving more favorable for printing 0-3 PZT composites. From depth/width–energy curves, optimal process parameters were determined. We designed and fabricated a soft piezoelectric metamaterial-based touch sensor using these parameters, achieving a customized output profile. This work offers critical insights into optimizing DLP for functional materials and expands the potential of 3D-printed piezoelectric composites.

## Introduction

Additive manufacturing (AM) allows for the assembly of structures with complex geometries and sophisticated material properties, exceeding the capabilities of traditional fabrication techniques and revolutionizing the way objects are conceived and created. It has become instrumental in designing and producing lightweight components, novel metamaterials, bio-inspired constructs, and multifunctional materials [1–3]. The AM of 0-3 type composites has attracted significant attention from researchers due to its capability for fabricating elaborate geometries [4]. Various AM techniques like vat photopolymerization [VPP; e.g., stereolithography (SLA) [5] and digital light processing (DLP) [6]], powder bed fusion [PBF; selective laser sintering (SLS) [7]], material jetting (ink jetting [8]), and material extrusion [fused deposition modelling (FDM) [9]] have enabled the creation of composite prints, using ceramic as filler and polymer as matrix.

Piezoelectric ceramics are essential functional materials in energy conversion, particularly in roles such as sensors, actuators, and transducers [10]. However, their innate brittleness and hardness restrict them to simple geometric forms, limiting their application scope. To address this limitation, piezoelectric composites, blending the piezoelectric property of ceramics with the moldability of polymers, have emerged as an adaptable solution [11]. Such composites can be used to produce more complicated structures by sintering to attain dense ceramic bodies or by preserving the polymeric matrix for direct use [7,12,13]. The unsintered composite combines the flexibility of polymers with the rigidity of ceramics, enhancing its applicability across various industries. Once activated through polarization, these composites exhibit piezoelectric properties that can serve multiple industrial needs, such as multi-dimensional deformation sensing [14], energy harvesting, integration of structure and function [4], soft robotics [15], and tissue engineering [16,17].

DLP is a light projection-based technology that manufactures complex shapes, renowned for its rapid production timelines, high resolution, and relatively uncomplicated machine maintenance. It has become a preferred method for creating ceramic–polymer composites in both 3-dimensional (3D) and 4D printing areas [18]. While DLP-based polymer fabrication is accelerating commercially, the development of ceramic–polymer composites compatible with DLP technology is progressing at a slower pace [10]. The major challenge is the physical and chemical property disparity between the fillers and the substrate, such as density and refractive index mismatch[19]. Density differences between ceramic particles and polymers make precipitation inevitable, disrupting the uniformity of the slurry and potentially hampering the light reactive polymer curing process, leading to print defects or substandard final products. Moreover, the different refractive indices of the filler and matrix in composites produce significant light scattering at their interfaces[20,21], which greatly exacerbates the complexity of the printing process and makes material development more difficult. Adequate cure depths are crucial for robust interlayer adhesion and guarantee material printability, while a minimal excess width is necessary to maintain print accuracy. Higher exposure energy can deepen the cure depth while unintentionally expanding the cured areas and affecting the surface finish. Fine tuning parameters such as energy dose, exposure time, and light intensity to meet both printability and accuracy requirements become tricky. Furthermore, a high solid content enhances the composite mechanical and piezoelectric properties but adversely influences light penetration, potentially resulting in incomplete curing and print failures. It is important to note that piezoelectric ceramics, such as lead zirconate titanate (PZT) and barium titanate (BTO), have higher refractive indices (2.66 [22] and 2.4 [23], respectively) compared to polymers (typically less than 1.6 [24]) and common ceramics like alumina and zirconia (with indices of 1.77 [25] and 2.17 [26], respectively). This characteristic typically results in a narrower printing process window, further complicating process optimization for DLP printing of piezoelectric ceramic–polymer composites.

Conventional approaches to minimize sediment caused by different densities include using dispersant addition [15] to increase spatial potential resistance and particle surface chemical modification [16] to change the hydrophilicity of particles by grafting chemical groups. These methods are hardly adequate due to severe experimental demands such as high temperatures and long durations. For optimizing the DLP printing process, common strategies aim to widen the process window, thereby simplifying the selection of printing parameters for piezoelectric composites. These involve implementing vacuum conditions during printing to promote polymerization by reducing oxygen interference [27] and formulating chemical mixes that optimize properties at lower filler contents [28]. But these methods often require costly materials, elaborate setups, or complicated chemical treatments while leaving the highly coupled parameters for high-filler-content material unresolved.

This study aims to systematically optimize the DLP printing of PZT–polymer composites to reduce material development costs and expand the application of DLP technology. For particle modification, a highly efficient plasma pretreatment approach is proposed to facilitate PZT functionalization before common chemical treatments. Plasma treatment activates the ceramic surface, enhancing functionalization in a short time, improving ink quality, and boosting the performance of the composite. For the DLP printing process, we focused on decoupling the process parameters through curing behavior analysis. We implemented an efficient exposure strategy, independently adjusting essential printing parameters, such as light intensity, exposure time, and energy dose, to investigate their effects on curing behavior and printability. Conventional DLP printing increases curing depth by raising light intensity to deliver more energy in the same exposure time, treating high-intensity and low-intensity exposures as equivalent. This study examines how varying light intensities affect curing depth under constant total energy, revisiting the mathematical model for DLP printing. A detailed analysis of key factors, including light intensity, energy dose, and photoabsorbers, guided the determination of optimal printing parameters, which were subsequently applied to fabricate a soft piezoelectric device with a complex meta-structure. These intricate structures are capable of generating custom voltage profiles, where each mechanical impulse induces dual signal pulses. This research is expected to be applicable to the novel 0-3 type composite for DLP printing and enlighten the functionality of the material, advancing structural metamaterials toward smart infrastructures in multimode sensing, soft robotics, and biomedical engineering fields.

## Results

### Plasma-assisted ceramic particle surface functionalization

To address the inherent bonding challenges between inorganic ceramics and organic polymers in 0-3 composites, where ceramic particles are dispersed as isolated inclusions within a continuous polymer matrix, a surface modification treatment was carried out to bridge the property gap. This process, known as particle functionalization, introduces new properties or functionalities by manipulating the functional groups on the particle surface [29]. This modification enhances the bonding between the polymer and ceramic, thereby improving the composite properties. The saline agent 3-(trimethoxysilyl) propyl methacrylate (TMSPM) [30] introduces acrylate surface groups to the powder, which crosslink with the matrix during curing, effectively anchoring the PZT to the polymer matrix and improving stress transfer and piezoelectric output.

However, because of the inert nature of ceramic surfaces, the modifying efficiency of ceramic surfaces is hardly enough. Adequate chemical modification often requires a long time and high reaction temperatures [31]. Plasma treatment is an effective method to increase the activity of the powder surface and improve the effects of modification without excessive chemical demands [32,33]. The plasma, an ionized gas replete with reactive species, including ions and electrons, can modify material surfaces effectively. For this study, we utilized an oxygen-activated plasma to append hydroxyl groups (-OH) [34] to the powder, rendering it more reactive and hydrophilic, which is favorable for subsequent surface modifications. With the activated particles, functionalization performance would be improved.

**Fig. 1.**
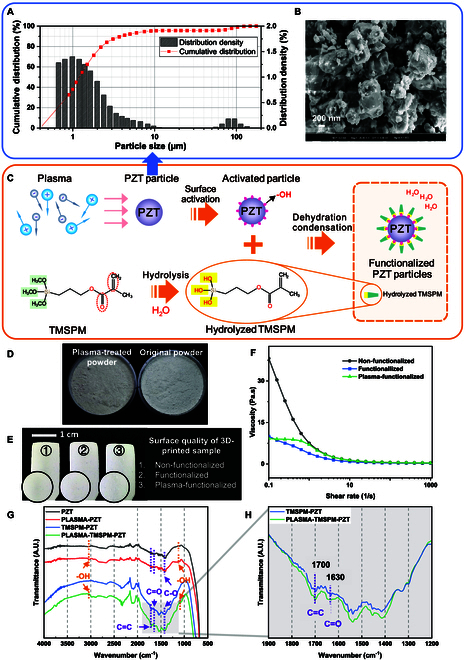
(A) Particle size distribution of PZT powder. (B) Surface morphology of PZT particles. (C) Schematic of plasma-assisted PZT ceramic particle surface functionalization process. (D) Appearance of the original PZT powder and plasma-treated powder. (E) Surface quality of 3D-printed samples with different treatments. (F) Viscosity of printing slurry with different treatments. (G) FTIR spectra of PZT powder with and without plasma treatment before and after functionalization. (H) Effect of plasma pretreatment on TMSPM graft modification on PZT powder.

We used commercial PZT powder for chemical modification as the filler for piezoelectric composites. The PZT filler size distribution (Fig. 1A) and morphology (Fig. 1B) of the ceramic particle were evaluated by a laser particle analyzer (HELOS-RODOS, Sympatec GmbH, Germany) and scanning electron microscopy (SEM; Merlin, ZEISS, Germany), respectively. The particle size distribution was in the range of 0.6 to 10 μm, and the mean size was 1.22 μm. The powder consisted of agglomerated particles with an irregular ball shape.

As shown in Fig. 1C, plasma-assisted surface functionalization activated the PZT particle surface and randomly formed the bonding of 2 phases [35] on the piezoelectric composite. After preheating (2 h) to dry the powder and reduce agglomeration, the plasma was activated with the power of 300 W, and surface treatment lasted for 30 min with stirring in the vacuum environment followed by a typical chemical functionalization process. Glacial acetic acid (1 ml) was dissolved in 9 ml of deionized water and mixed with 2 ml of TMSPM in 48 ml of ethanol. After the completion of the coupling agent hydrolysis reaction, 20 g of pretreated PZT was added and stirred at 80 °C for reflux for 4 h. The silane coupling agent was grafted onto the ceramic surface through the dehydration condensation reaction between the hydroxy groups -OH on the particle surface and the hydrolyzed coupling agent. Finally, the TMSPM-grafted PZT powder was cleaned and dried. The critical functional groups (C=C and C=O) on the saline are emphasized in the formula (red dot circle). Fourier transform infrared spectroscopy (FTIR) with ATR (attenuated total reflection) accessory (Vertex 70v, Bruker, Germany) was hired to identify the grafted functional groups on the powder surface to evaluate the performance of plasma pretreatment and surface functionalization.

Figure 1D illustrates the result of the plasma treatment before functionalization. After exposure to a high-energy plasma blast, the plasma-treated particles looked slightly darker than the original powder. Ceramic composite pastes were prepared by mixing powders with various treatments and resins. Rheometer testing (Discovery HR-30, TA Instruments, USA) revealed that the treatments significantly influenced the viscosity of the pastes (Fig. 1F). The chemical modification of the powder notably improved the rheological properties of the slurry, reducing viscosity from approximately 40 Pa·s to 8 Pa·s at a shear rate of 0.1 s^−1^ by minimizing friction and resistance between particles [36]. However, plasma treatment caused a slight increase in viscosity, raising it to around 9 Pa·s. This may be due to the introduction of new functional groups by the plasma treatment, which increased intermolecular interactions. The hydrophilic functional group -OH can cause a sudden rise in slurry viscosity at high solid content levels [37].

Figure 1E shows the surface quality of printed parts subjected to different treatment processes. The unmodified samples (1) exhibited a smoother surface finish compared to the modified samples (2) and (3). The rougher surface in the modified sample (3) is attributed to the plasma-assisted modification, which activates the ceramic powder surface and may induce unexpected chemical reactions.

FTIR results in Fig. 1G are divided into modified (TMSPM-PZT and PLASMA-TMSPM-PZT) and unmodified groups (PZT and PLASMA-PZT). Unlike unmodified PZTs, modified PZTs possess peaks at 1,700 and 1,630 cm^−1^ corresponding to the functional groups of C=C and C=O stretching vibration [27,28]. These peaks represent the methacrylate surface groups on TMSPM and show that the TMSPM and PZT particles have good chemical and interfacial connections. Comparing PZT (red) and PLASMA-PZT (black) in the unmodified group, the peak at 3,050 and 1,200 cm^−1^ on the PLASMA-PZT indicates that additional -OH was added to the PZT powder by plasma [38]. These -OH on the surface would improve the activation of the particle and promote the subsequent chemical reactions. Figure 1H shows that the plasma-treated powder leads to better modification. The intensity of crucial characteristic peaks (C=O and C=C) on the TMSPM of PLASMA-TMSPM-PZT was stronger than the no-plasma version when they shared baselines around peaks in the spectra. This is due to the fact that under the same chemical treatment conditions, the plasma pretreatment first attached the -OH functional group onto the powder surface, resulting in improved coupling agent grafting. After the functionalization process, some of -OH groups were still observed on the PLASMA-TMSPM-PZT compared to TMSPM-PZT sample, which could rise the viscosity in slurries pretreated with plasma. These FTIR results proved that plasma pretreatment is highly effective in enhancing the chemical grafting efficiency on the ceramic powder surface. The effect of plasma pretreatment on the piezoelectric and mechanical properties of the composites will be further discussed in the following paragraphs.

### Investigating the curing behavior with different exposure strategies

The cure behavior (cure depth and excessive width) of the material is closely related to its printability and printing accuracy. Shallow curing depth makes adjacent layers difficult to combine tightly and makes printing hard, while large excessive width is out of design dimensions and significantly impacts printing accuracy. Analyzing cure behavior is a critical part of optimizing the printing process. Here, we employed an efficient exposure strategy, separately controlling the light intensity and energy dose to study their respective effects on the cure behavior of PZT composites in order to help decouple the DLP printing parameters. These exposure strategies allow for the collection of multiple data points in a single experiment, eliminating errors caused by repeated experimental setups. By adjusting individual parameters, multiple sets of comparative experimental data can be quickly obtained, improving experimental efficiency.

**Fig. 2.**
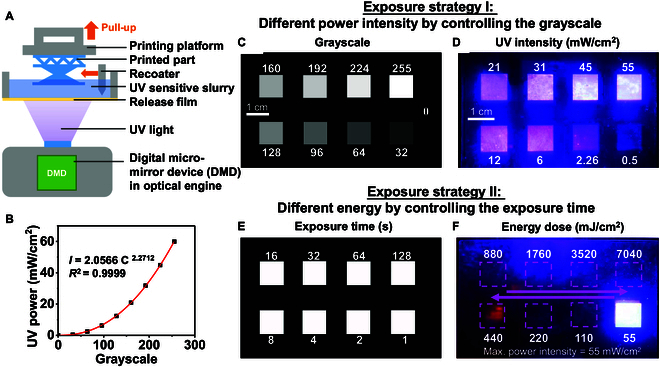
(A) Schematic of the DLP printing technology. (B) Relation between grayscale of light craft and measured UV power. (C) Exposure strategy I: different level of grayscales during one exposure and its (D) corresponding measured UV power intensity. (E) Exposure strategy II: with different exposure time and (F) accumulate energy by sequence when measured energy intensity is 55 mW/cm^2^ (rated power = 60 mW/cm^2^, grayscale = 255).

Figure 2A demonstrates a schematic of the bottom-up DLP 3D printer used in this study. Bottom-up 3D printing is favorable due to its effective use of printing materials, and the combination of a light engine with a digital micro-mirror device (DMD) permits high light power intensity and high-resolution output simultaneously. For high particle loading printing, where the viscosity of the printing ink is high, a recoater is equipped to ensure that a smooth and even layer of material is applied before each curing step. A bottom-up DLP printer (Prism M3, 3KU Tech., China) was hired to develop piezoelectric composites. The DLP 3D printer is furnished with a light engine with a 405-nm wavelength ultraviolet (UV) light source and 60 mW/cm^2^ rated light intensity. The printer resolution in the vertical (*z*) direction is 10 μm, determining the minimum layer thickness.

The optical machine is gamma-corrected to ensure the uniformity of the output brightness, so the intensity and brightness of the display are not linear. The following equation can describe this relation [39]:

$$I={\mathrm{aC}}^{\gamma }$$ (1)

where I is the measured light intensity output (UV power, mW), C is the digital input from the optical engine (brightness, grayscale), γ is the correction factor of the optical engine, and a is an experimental constant. Figure 2B reveals the nonlinear relation between the grayscale of the optical engine and measured UV power. UV power was measured by a UV energy meter (LS133, Linshang Tech, China). The γ factor of this optical engine in the printer was 2.2712.

The relationship between grayscale and light intensity is nonlinear, allowing precise control over the incident light intensity during printing. By adjusting the grayscale of the projection pattern, it is possible to achieve various printing effects or facilitate ultrafast printing [40]. Figure 2C to F introduces a series of exposure strategies that manipulate light intensity and exposure time (control energy dose) separately for the cure behavior study. For exposure strategy I (Movie S1), different UV powers were implemented in a single exposure by controlling the grayscale [41,42] (Fig. 2C). The range of UV output was paired by 8 gray levels so that the whole UV power range of the machine was investigated at a single exposure. Because the γ value corrected the optical machine, the output UV power corresponding to the equal interval grayscale is nonlinear. The measured power intensity ranges from 0.5 to 55 mW/cm^2^ (Fig. 2D). Within a single exposure process, the same exposure energy was applied to every sample with varying intensity and time (E = I * t). Then, different exposure energy doses were implemented by repeating the exposure processes. Five energy doses were utilized to observe the cure behavior for different powers in Table 1. A power intensity of 0.5 mW/cm^2^ (grayscale = 32) was too weak to cure a composite material, so 7 power intensities in total were tested.

**Table 1.** Parameters for studying curing behavior when energy dose is constant, and power intensity is different using exposure strategy I

No.	Weight fraction (%)	Power intensity (mW/cm^2^)								Energy dose ^a^ (mJ/cm^2^)	Photoabsorber
		55	45	31	21	12	6	2.3	0.5		
		**Exposure time (s)**									
1	50	1	1.3	1.9	2.9	4.8	9.6	24.4	-	~57	-
2	50	2.5	3.3	4.6	7.1	11.9	23.5	60.0	-	~130	-
3	50	6.1	8.1	11.4	17.4	29.3	57.9	147.7	-	~287	-
4	50	14.9	19.9	28.1	42.8	72.1	142.4	363.4	-	~641	-
5	50	36.7	49	69.2	105.3	177.4	350.3	894.3	-	~1,441	-

^a^ The energy dose is an approximation because the output light energy is unstable during a long exposure. Measured energy was used in the data analysis section.

Exposure strategy II (Movie S2) aimed to uncover the curing behavior of the material at a fixed power by modifying exposure time. For example, when the power intensity was 55 mW/cm^2^ (grayscale = 255), the exposure time series (from 1 to 128 s) was used to adjust the energy dose of curing (Fig. 2E). The incident energy accumulated (from ~55 to 7,040 mJ/cm^2^) with the increase in exposure time as shown in Fig. 2F. The cure behavior of different high loading contents of ceramic at 50, 60, 66.7, 71.4, and 75 wt % (the weight ratio of ceramic powder to polymer matrix = 1:1, 1.5:1, 2:1, 2.5:1, and 3:1, respectively) was tested by exposure strategy II (Table 2). Slurry with different solid contents has different sensitivity to UV light, so the energy of each group of experiments was varied to limit excessive exposure and avoid interference between samples. However, when the data were analyzed, all the measured data were aligned according to actual energy. Movies S1 and S2 demonstrate these different exposure strategy processes with transparent commercial resin.

**Table 2.** Parameters to studying curing behavior for different ceramic contents and the photoabsorber when power is constant (55 mW/cm^2^) using exposure strategy II

No.	Weight fraction (wt %)	Exposure time (s)								Maximum energy ^a^ (mJ/cm^2^)	Photoabsorber
1	50	0.5	1.5	3	8	18	44	105	-	~5,775	-
2	60	1	3	7	16	37	89	211	-	~11,605	-
3	66.7	1	3	7	16	37	89	211	-	~11,605	-
4	71.4	1	3	7	16	37	89	211	500	~27,500	-
5	75	1	3	7	16	37	89	211	500	~27,500	-
6	50	1	3	7	16	37	89	211	-	~11,605	Sudan IV

^a^ The maximum energy is an approximation because the output light energy is unstable for a long exposure. Measured energy was used in the data analysis section.

Different exposure strategies corresponded to different key printing process parameters, simplifying the parameter testing process and reducing errors caused by sample duplication and repeated experiments. This approach improved both experimental efficiency and accuracy.

The ceramic slurry was poured and recoated evenly on a transparent film. After implementing the designed exposure strategies (Tables 1 and 2), the cured samples attached to the film. During a photocuring printing process, curing depth ($C_{d}$) and excess width ($W_{\mathrm{ex}}$) of the cured samples must be thoroughly studied to evaluate the cure behavior and mechanism of ceramic composite slurry, which is crucial to improving polymerization quality and accuracy.

**Fig. 3.**
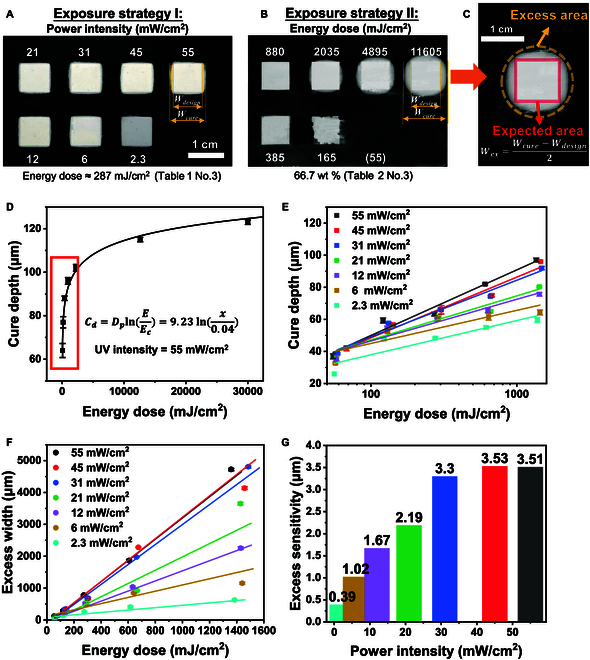
Appearance of excess widths for (A) exposure strategy I and (B) II (Table 1, No. 3, and Table 2, No. 3, respectively). (C) Excess and expected area of cured sample. (D) Cure depth curve for 50 wt % slurry fits quasi-Beer–Lambert law. (E) Curing depth and (F) excess width with increasing exposure energy for different exposure intensity. (G) UV intensity sensitivity of excess width for different exposure intensity.

The curing depth $C_{d}$ is defined as the maximum polymerization distance of a ceramic suspension under UV light exposure [43]. The excess width $W_{\mathrm{ex}}$ refers to the cured width extending beyond the incident light width [44]. $C_{d}$ varies with different exposure parameters and was measured using a high-precision screw micrometer equipped with a force-measuring device to ensure measurement accuracy for fragile samples. The thickness of the sample and film was measured directly, and the depth of cured samples was evaluated by subtracting the thickness of the film, which was constant. Meanwhile, $W_{ex}$ was measured under an optical microscope and defined by the following equation (Fig. 3C):

$$W_{\mathrm{ex}}=\frac{W_{\text{cure}}-W_{\text{design}}}{2}\;$$ (2)

where $W_{\text{design}}$ = 9 mm in this study to avoid the dependence on sample size and curing behavior [45]. When increasing the incident energy, deepened cure depth is favored for interlayer bonding, but the excess width must be minimized to improve the precision and surface finish of printing.

Additionally, pigments were introduced as photoabsorbers [46,47] to refine the printing quality. Sudan IV pigments (0.1 wt %) were added to the slurry. The cure depth and the excess width of the shading agent-added material were measured in the same way as the original formulation.

### Cure behavior for piezoelectric composite

#### The influence of different light intensity and energy doses on cure behavior

After exposure, the appearance of excess area of cured samples for exposure strategy I and II is shown in Fig. 3A and B. The excess (dotted yellow line) and expected (solid red line) areas can be identified easily in Fig. 3C. The excess width is deduced using Eq. 2. For the case in Fig. 3A, despite identical energy dose levels, increased power intensity led to wider excess areas. A similar trend appeared in Fig. 3B, but with more noticeable differences due to larger energy gaps between the samples, indicating that excessive area is related not only to total energy but also to power intensity. The excessive phenomenon will be quantified to investigate this issue.

Figure 3D demonstrates the relationship between curing depth and energy dose when the UV intensity is at its peak capacity (55 mW/cm^2^), following the quasi-Beer–Lambert law in Eq. 8. That is, cure depth exhibited a logarithmic increase with incident energy. Figure 3E shows that the relationship between cure depth and energy under each light intensity approximates the Beer–Lambert correlation. With the same incident energy (5 groups of energy dose in this experiment), higher light intensity results in a deeper curing depth. The slope and *x* intercept of the related curve vary with the incident light intensity, differing from the SLA process. These results are interpreted in the section of theory on cure behavior for DLP printing. One brief explanation is as follows. Photoinitiators generate free radicals by absorbing energy. When more energy is supplied to the composite exposed surface, it allows for greater energy absorption by the photoinitiators, enhancing the cure depth [48]. Therefore, higher incident light intensity is beneficial for 0-3 type composites, as the curing depth for these materials is difficult to increase due to scattering, making the printing process more challenging. In SLA, the depth of cure is determined only by the incident energy, independent of the incident light intensity. Slope and *x* intercept of the related curve refer to the penetration depth ($D_{p}$) and critical energy ($E_{d}$) in Eq. 8, which are constant material parameters.

By studying the polymerization in the horizontal direction, a quasi-Beer–Lambert relationship was used for the broadening phenomenon [44].

$$w_{\mathrm{ex}}=S_{w}\text{ln}\left(\frac{E_{0}}{E_{w}}\right)\;$$ (3)

where $S_{w}$ is the width sensitivity and $E_{w}$ is the width critical energy.

In DLP, excess width varies for energy dose, and both the width critical energy dose and width sensitivity are affected by light intensity [45,49,50]. The trend of excess width, resembling cure depth expansion with increased energy, is highlighted in Fig. 3F. Also, since the maximum energy in this experiment was only about 1,500 mJ/cm^2^, the logarithmic and linear relationships were closer at lower energies. Higher energies will be implemented in the following section.

Comparing Fig. 3E and F, excessive width is more sensitive to changing light intensity than curing depth, with ranges of ~10-μm and ~1,000-μm scale, respectively. However, curing depth is critical to ensuring the material's printability. Therefore, it is reasonable first to maintain the required curing depth and then control the excessive width. Higher light intensity helps meet this requirement. Figure 3G introduces excess sensitivity as the gradient of the linear fit between excess width and energy dose. Although both the excessive width and cure depth increase with higher light intensity, the increment rate of excess width (excessive sensitivity) is dampened. When the light intensity increases, the curing depth increases, but the sensitivity to excessive width no longer rises. At this point, it becomes feasible to control the excessive width, for example, using light absorber.

#### The influence of loading contents and photoabsorber on cure behavior

**Fig. 4.**
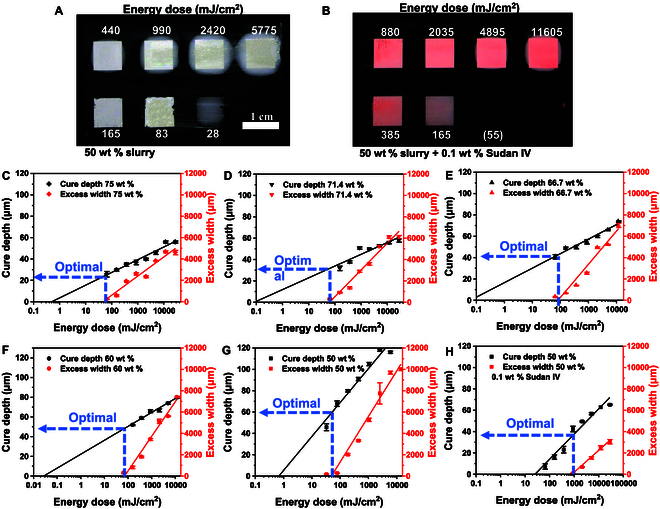
Appearance of excess width for 50 wt % composite slurries (A) without and (B) with 0.1 wt % Sudan IV as photoabsorber (Table 2, Nos. 1 and 6). (C to G) Cure behavior of different ceramic contents, 50, 60, 66.7, 71.4, and 75 wt %. (H) Cure behavior of 50 wt % with 0.1 wt % Sudan IV.

Utilizing pigment as a photoabsorber can control the light absorption capabilities of the material by dampening scattering [47]. Specifically, a pure polymer ink solution containing yellow (Tartrazine) and red (Sudan IV) pigments was found to yield superior printing results when the printing wavelength was set at 405 nm [46]. As shown in Fig. 4A and B, without the photoabsorber (0.1 wt % Sudan IV), the excess area was significantly pronounced, but when introduced, the excess area was substantially reduced. For the absorber-added material, a higher energy of 55 mJ/cm^2^ did not cure the lower right corner sample, unlike the original material, which cured at 28 mJ/cm^2^. This fact indicates that light absorbers significantly reduce UV light sensitivity in the material. With lower energy (<2,000 mJ/cm^2^), the broadened area was hardly noticeable to the naked eye, as reflected in Fig. 4B.

Moreover, the depth of critical energy $E_{d}$ with an inhibitor exhaustion model based on the assumption that $E_{d}$ is the dose necessary to exhaust the polymerization inhibitors [51] is presented by:

$$E_{d}=\left(1-\phi \right)\frac{\mathrm{h\nu}}{\Omega }\left(\gamma_{\mathrm{inh}}c_{\mathrm{inh}}+\gamma_{D}c_{D}\right)\frac{1}{\varepsilon_{P}c_{P}}\;$$ (4)

where hν is the photon energy, Ω is the number of free radicals produced per photon absorbed, $\gamma_{\mathrm{inh}}$ is the number of radicals removed per inhibitor, $\gamma_{D}$ is the number of radicals that were not generated as an inert dye absorbed photon, $\varepsilon_{P}$ is the photoinitiator extinction efficiency, and $c_{\mathrm{inh}}$, $c_{D}$, and $c_{P}$ are the concentration of the inhibitors, dye, and photoinitiator, respectively. In this experiment, $E_{d}$ increases with the introduction of color ($\gamma_{D}c_{D}$), and more energy is required to cure the paste, weakening the curing and scattering simultaneously.

According to the conclusions drawn in the preceding section, a higher light intensity has been identified as advantageous for the printing process to keep a deeper cure depth and control excessive width. Moreover, for the same energy requirement, using a higher light intensity results in faster print speeds. Consequently, the subsequent experiments employed the maximum light intensity of the light machine (55 mW/cm^2^). Figure 4C to G illustrates the curing behaviors for various ceramic contents (50, 60, 66.7, 71.4, and 75 wt %), illustrating that higher solid content posed increasing difficulty in curing the material. The cure depth and excess width curves facilitate the determination of an optimal curing point. This point, denoted by the *x* intercept of the excess width fitted line, indicates no excess width. The corresponding curing depth at this point is regarded as the optimal depth (~20, 30, 40, 50, and 60 μm for varying material contents, respectively), with the printing layer thickness set at half of this value. This allows incident light to penetrate through each 2 layers to strengthen the interlayer connection at each exposure.

In addition, the previous finding reveals a potential preference for higher intensity to improve print quality. Therefore, optimal exposure energy is critical in determining the appropriate exposure time. Notably, the optimal energy ($E_{w}$ critical energy for width direction) was found to be in the range of 50 to 100 mJ/cm^2^ for the various material compositions in this experiment without significant broadening. This dose suggests that ~2 s can be used to achieve effective printing for each material type. However, introducing color changes this energy, as shown in Fig. 4H, where the optimal energy increases to approximately 800 mJ/cm^2^. Consequently, this adjustment leads to an increased optimal exposure time of 14.5 s, but at the cost of reducing the cure depth from 60 to 38 μm.

Comparing Fig. 4G and H, the addition of absorbent significantly increased the critical curing energy in both depth ($E_{d}$) and width direction ($E_{w}$), also reducing the light sensitivity of the material referring to the gradient of the fitted curve ($D_{p}$ and $S_{w}$). The critical curing energy $E_{d}$ of material was increased from 500 to 1,000 mJ/cm^2^, while the cure depth decreased from 60 to 38 μm (Fig. 4G and H). Although the curing depth and excessive width have decreased, the cure depth is still greater than the *z* axis resolution of the machine (10 μm), meaning printing is still feasible. In other words, by adding a light absorber, printing precision is improved as the excessive area is eliminated, and the curing depth still meets the minimum requirements for printing. This makes it easier to adjust excessive width with a reasonable cure depth. The addition of light absorbers is an effective way to change the printability of a material. Further experiments will be conducted to evaluate printability and printing accuracy using the disclosed printing parameters in the next section.

### Discussion of cure behavior theory in DLP 3D printing

The differential form of Beer–Lambert law explains how light intensity changes with the increasing distance from the incident surface,

$$\frac{\mathrm{dI}\left(z\right)}{\mathrm{dz}}=-\mu I$$ (5)

where I is the light intensity, z is the distance from the surface of the slurry, and μ is the attenuation coefficient, which is typically considered constant for a specific material when ignoring the variations that occur during the phase transition in material curing. Integrating both sides of Eq. 5 yields $I\left(z\right)=I_{0}e^{-\mu z}$, where $I_{0}$ is the incident intensity at the surface.

SLA and DLP, both photopolymerization-based 3D printing technologies, differ significantly in light intensity and exposure time. In SLA, the incident light intensity is extremely high (~10^7^ mW/cm^2^), with a very short exposure time (~10^−5^ s), making energy absorption nearly instantaneous compared to the curing timescale (~1 s) [52,53]. This allows the attenuation coefficient μ to be treated as constant during exposure. In contrast, DLP operates at much lower light intensity (~10 mW/cm^2^), requiring significantly longer exposure times (~1 to 10 s) for energy accumulation and curing.

The extended exposure time in DLP makes variations in μ nonnegligible [48,53,54], which can be attributed to 2 possible factors. First, the duration of DLP printing is comparable to the curing timescale, leading to strong coupling between light penetration (scattering) and the curing process (photoinitiator consumption). Second, the phase transition of the material from liquid to solid during curing also influences the attenuation of light.

This change in μ during the curing process makes it dependent on both light intensity and exposure time. To simplify the mathematics and achieve an analytical solution, we treat μ as an average value over the entire curing process in the subsequent mathematical model. Consequently, when applying Eq. 5, μ is still treated as a constant but inherently reflects its dependence on light intensity and time.

For photopolymers, the extent of the reaction depends upon the light energy absorbed per unit volume. Instead of the energy absorption per unit area in previous models, denoting the absorption energy per unit volume by $U\left(z\right)$ during the exposure time $t_{e}$, which is equal to the decrease of the light intensity per unit depth:

$$U\left(z\right)=-\frac{\mathrm{dI}\left(z\right)}{\mathrm{dz}}t_{e}=\mu I_{0}e^{-\mu z}t_{e}$$ (6)

Denoting $U_{d}$ the threshold of the energy absorption density (per unit volume) required for polymerization at the depth $z=C_{d}$, using Eq. 6, one has $U_{d}=\mu I_{0}t_{e}e^{-\mu C_{d}}$, which yields

$$C_{d}=\frac{1}{\mu }\text{ln}\left(\frac{I_{0}t_{e}}{U_{d}/\mu }\right)$$ (7)

In prior SLA studies, $I_{0}t_{e}$ is commonly defined as the incident radiance energy density and denoted by $E_{0}$ (with a unit of energy per unit area), $U_{d}/\mu$ is denoted by $E_{d}$ (in unit of energy per unit area), and $\frac{1}{\mu }$ is defined as a material parameter called penetration depth $D_{p}$. Equation 7 can be written as the known expression [55]:

$$C_{d}=D_{p}\text{ln}\left(\frac{E_{0}}{E_{d}}\right)\;$$ (8)

If one uses Eq. 8 to study the curing process in DLP, curing depth $C_{d}$ will be independent from intensity $I_{0}$ if $E_{0}=I_{0}t_{e}$ remains constant. However, our experimental data reveal that $C_{d}$ is dependent on intensity $I_{0}$.

**Fig. 5.**
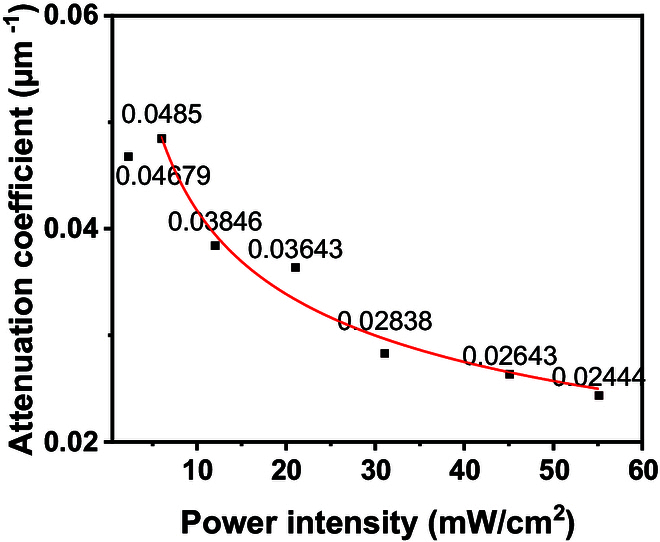
Attenuation coefficient obtained from fitted curve of cure depth with different light intensity and constant incident energy dose.

As shown in Fig. 3E, within testing range, the curing depth increases with increasing light intensity when the incident energy remains constant. Figure 3E also illustrates the fitted curves of cure depth data under different light intensities. When the *x* axis is logarithmic, the fitted curve appears linear, and the slope of the curve varies significantly under different light intensities. Let $E_{1}$ =1 mJ/cm^2^ (unit of $E_{0}$), then Eq. 7 can be rewritten as $\begin{array}{c} C_{d}=\frac{1}{\mu }\text{ln}\left(\frac{I_{0}t_{e}E_{1}}{E_{1}U_{d}/\mu }\right)=\frac{1}{\mu }\text{ln}\left(\frac{I_{0}t_{e}}{E_{1}}\right)+\frac{1}{\mu }\text{ln}\left(\frac{E_{1}}{U_{d}/\mu }\right) \end{array}$. Here, $\frac{1}{\mu }$ is the slope and $\frac{1}{\mu }\text{ln}\left(\frac{E_{1}}{U_{d}/\mu }\right)$ is the intercept of the fitted curve, respectively, in Fig. 3E. Based on the experimental cure depth results, the average attenuation coefficient μ and $U_{d}$ for each light intensity can be obtained. Figure 5 shows the average attenuation coefficients under different light intensities at the same total energy. The fitted curve function for the attenuation coefficient is given as $y=0.0818x^{-0.3494}+0.005$, and $U_{d}$ is fitted and averaged to be approximately 0.028 mJ/cm^3^ for this composite. Here, 0.005 ${\mathrm{\mu m}}^{-1}$ approximately represents the initial attenuation coefficient of the composite slurry $\mu_{0}$. Within the experimental range (2.26 to 55 mW/cm^2^), at constant incident energy density, the average attenuation coefficient decreases with increasing incident light intensity, while the curing depth increases.

### Printing performance of piezoelectric composites and structures

**Fig. 6.**
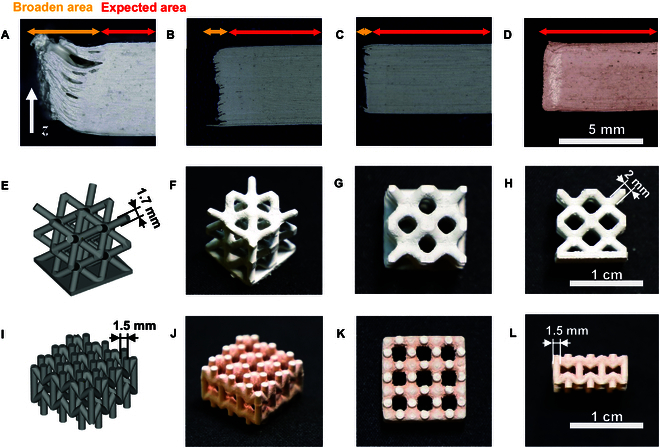
Section views of printed parts under the microscope when the exposure time is (A) 6 s, (B) 4 s, and (C) 2 s with 20-μm layer thickness, and (D) 10 s with 15-μm layer thickness. (E) Designed and (F to H) printed BCC structure. (I) Designed and (J to L) printed auxetic array structure.

In the previous section, the optimal printing parameters for the 50 wt % PZT composites were determined (layer thickness of 30 μm, ~50 to 100 mJ/cm^2^, ~2 s). Optimal parameters point out the position of the process window for the current material. On this basis, the print parameters can be tuned to suit the different requirements. For example, if print efficiency is favored, then the exposure time can be slightly shortened on the optimized parameters to obtain faster print speeds, but at the expense of some mechanical property. In this experiment, higher energy and thinner layers (layer thickness of 20 μm, with exposure times of 2, 4, and 6 s per layer) were chosen to ensure interlayer bonding and the mechanical property of the printed parts, and then the effect of the printing parameters was examined. The printing performance of various parameters and structures is depicted in Fig. 6. Prolonged exposure time (Fig. 6A, at 6 s) led to substantial excess width, notably with unpolymerized slurry trapped between layers in the section view. Reducing the exposure times in Fig. 6B and C yielded gradual improvements in dimensional accuracy along the *z* axis and minimized excess width. Although printing is still possible with extended exposure times (4 and 6 s), the significant broaden areas result in poor printing accuracy and surface finish directly. In contrast, upon introducing a photoabsorber (Fig. 6D), consistent with previous results from the working curve (19 μm, 800 mJ/cm^2^, ~14 s for 50 wt % + 0.1 wt % Sudan IV, in Fig. 4H), the 15-μm layer thickness printed with a longer exposure of 10 s maintained sharper boundaries without excessive width.

Figure 6E to L compares designed versus printed structures [body-centered cubic (BCC) and auxetic arrays, respectively]. Structures printed with a photoabsorber (Fig. 6I to L) accurately matched the intended 1.5-mm rod dimensions, unlike their counterparts without the absorber (Fig. 6E to H), which slightly exceeded the design specifications. These results confirm that photoabsorbers can improve printing precision and enable the creation of refined features, although some surface roughness remains, suggesting that further process refinement is required in future research.

### Property of 3D-printed soft piezoelectric composite and sensing element

**Fig. 7.**
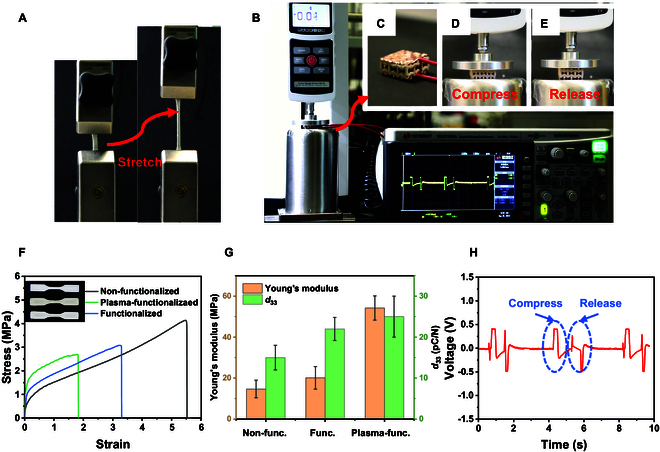
(A) Stretched and (B) compressed unit of soft piezoelectric material. (C) Representation of the assembled piezoelectric sensing element. (D) Compress and (E) release state of piezoelectric structure. (F) Mechanical property of functionalized, plasma-functionalized, and nonfunctionalized piezoelectric composite and (G) their average Young’s modulus and piezoelectric coefficient *d*_33_. (H) Electrical response of 3D-printed soft piezoelectric meta-structure.

To probe the sensor capabilities, a custom testing setup was assembled, comprising a universal testing machine (ESM303, MARK-10, USA), a digital storage oscilloscope (model EDUX1002A, Keysight Technologies, USA), and a standard signal amplification circuit. Conductive copper tapes encased the top and bottom surfaces of the specimens to serve as electrodes, thereby facilitating the transmission of electrical signals through connected wires. The mechanical gauge integrated into the universal testing apparatus facilitated precise and consistent control of the compression force (90 N, 100 mm/s). As depicted in Fig. 7A, the tensile test was implemented for 3 times and reveals the stretchability of the 3D-printed piezoelectric composite.

Moreover, an amplifier systematically magnified the tiny signals generated by the compression force acting upon the sensing element. During each compression cycle, the oscilloscope collected and documented the magnified signals. Simultaneously, incidental signals generated by finger imprints were also recorded.

Figure 7F shows the typical stress–strain curves of the piezoelectric composites under different treatment processes. Their average Young's modulus and piezoelectric coefficients can be found in Fig. 7G. These figures illustrate the advantages gained through a functionalization process that enhances the bond between the composite 2 phases, improving overall properties. An analysis of the tensile stress–strain relationship indicates a higher Young’s modulus in the plasma-treated sample (54.22 ± 4.3 MPa), surpassing that of both the conventionally treated sample (20.11± 5.5 MPa) and the untreated version (14.67 ± 5.9 MPa). Although the enhanced Young’s modulus resulted in reduced ductility, with elongation of 5.5% for untreated samples against 3.5% and 1.8% in the conventionally treated and plasma-treated samples, the remaining flexibility still meets the demands of several engineering applications. Furthermore, in the Fig. 7G, the functionalization process not only rises the mechanical property of the composite but also improves the piezoelectric performance, elevating $d_{33}$ from 15 ± 3 pC/N to 22 ± 4 pC/N. Therefore, plasma treatment for the powder not only improves the quality of the printing ink but also increases the mechanical and piezoelectric properties of the composite.

The configuration of the soft sensing element assembly, featuring wire-connected electrodes made of conductive tapes, is depicted in Fig. 7C. Upon the immediate application of a periodic force (90 N) to the sensor surfaces, a series of signals is promptly detected. As illustrated in Fig. 7H (Movie S4), each force impulse generates 2 sequential pulse signals: The first pulse indicates compression (Fig. 7D), and the second marks the release process (Fig. 7E). These dual signal pulses arise from the specific structural design and the inherent flexible mechanical properties of the sensing element. In contrast to conventional solid piezoelectric sensors that typically produce only one signal pulse per force impulse, the observed multiset data indicate the multiple states of joints within the hollow structure. During compression, node contact alters the electric displacement field, initiating the first signal set (Fig. 7D). During release, node separation changes this field again, creating a second signal set in Fig. 7E (see also Movie S3). The feedback signal mirrors that of conventional piezoelectric sensors in situations where displacement is too small to cause node contact, as cited in [9,56,57]. For instance, in cases where a finger gently taps the sensor, it generates only a single set of signals for each tap, as demonstrated in Movie S5. The ability to attain multiple states through structural design underscores the potential for creating and fabricating flexible sensors that may accommodate diverse modes.

## Discussion and Conclusion

In conclusion, this study addressed the phase mismatch challenges in 0-3 PZT composites for DLP printing by optimizing the process through particle pretreatment and a comprehensive analysis of curing behavior, combining both experimental and theoretical approaches.

PZT particles were pretreated with high-energy plasma to address the sedimentation issues caused by density difference. FTIR results indicated that the -OH groups added to the ceramic surface by the plasma treatment benefited the subsequent TMSPM grafting, enhancing the chemical modification and composite property. The intensity of the characteristic peaks of the coupling agent on the plasma-treated powder was greater than on the untreated powder. This treatment activated the powder surface, promoted chemical modification, reduced sedimentation, and facilitated printing ink preparation. This study verified the promoting effect of plasma pretreatment on chemical functionalization, but further research is required to optimize treatment parameters, such as activation power and treatment time, for improved results.

We tested key DLP printing process parameters, such as light intensity and energy dose, using different exposure strategies to assess the curing behavior of the composite and examine how these parameters affect printability and accuracy. Based on the results, we developed quantitative parameter guidelines and formed a ready-to-use process datasheet. This practical datasheet defines the process window for the current material, enabling parameter adjustments to meet specific requirements, such as optimizing printing speed or enhancing part strength. While the approximate location of the narrow process window is identified, fine-tuning of parameters and experimental testing are still crucial for achieving higher-precision printing. Additionally, incorporating a light absorber can increase the critical curing energy and reduce the material's light sensitivity, making parameter adjustments easier and further improving print accuracy. This provides an effective method for enhancing print precision.

This study introduced a curing depth model specifically for DLP printing, distinguishing it from SLA and offering new insights into the photopolymerization process. Due to the differing timescales of curing, the attenuation coefficient in DLP printing is no longer a constant. Through theoretical analysis and experimental measurements, we show that although both SLA and DLP are photopolymerization processes, their curing behaviors and mathematical models differ due to the different timescales of the physical curing processes. Model for DLP explains the experimental phenomena under varying light intensities, but due to the light power limitations of our experimental equipment, further studies with higher light intensities can be conducted in the future.

This study systematically optimized the major step in the DLP printing process. A meta-structure-based soft piezoelectric touch sensor was designed and fabricated, producing a customized voltage profile in response to force impulses. This work advances the optimization of DLP printing parameters and the exploration of curing behavior for novel functional materials. The optimization, compatible with common commercial DLP machines, facilitates the efficient printing of smart structures with complex architectures. This is a new perspective to designing structural-functional-integrated composites for DLP printing, which allows the structures to act as built-in sensors, providing customized signals without requiring additional sensor attachments. 3D-printed piezoelectric composites have the potential to revolutionize fields such as energy harvesting for wearable devices and infrastructure monitoring, where piezoelectric materials convert mechanical vibrations into electricity to power sensors and systems. Additionally, it holds promise for medical devices, such as implantable sensors and therapeutic systems, as well as in aerospace and consumer electronics for smart components, haptic feedback systems, and flexible electronics.

## Materials and Methods

### Ceramic slurry preparation

The commercial PZT-5H (Hongsheng Acoustics, China) ceramic powder (piezoelectric coefficient *d*_33_ = 600 pC/N and a relative dielectric constant ε = 3,000) was chosen as the filler of the piezoelectric composite. The liquid light reactive photopolymer contains the photoinitiator (1 wt %), highly active dilution acrylate monomer (33.3 wt %) for better fluidity, and urethane acrylate oligomer (65.7 wt %) for flexibility. The ceramic–polymer suspensions comprise as-prepared ceramic powder (50, 60, 66.7, 71.4, and 75 wt %), photopolymer (balance), defoaming agent (0.2 wt %), and the dispersant (1 wt %). The photoabsorber (Sudan IV, 0.1 wt %) was added optionally. They were mixed using a high-speed cantilever agitator (Huxi, China) with a dispersion disk at 1,200 rpm for 60 min for dispersion. Then, the suspension was degassed on a vacuum-deforming container for 30 min.

### Polarization of piezoelectric material

To maintain the flexibility from polymer, sintering is not needed in this study. Poling is implemented after printing directly. Due to the mismatch of the dielectric constant between polymer (~5) and PZT piezoelectric ceramic (~3,000), poling the piezoelectric composites is difficult. This disparity results in only a lower voltage being applied to the piezoelectric phase [58–60]. To overcome this issue, a modified poling method [61] was employed with a poling machine (HYJH-FL-30 kV, Huiyan Tech, China) as the polarization technique for the PZT composite to enhance the poling voltage and efficiency. This method allowed for a maximum applied electric voltage of 220 kV/cm for 1 h at room temperature, significantly improving the poling of 3D-printed ceramic polymer composites.

### Mechanical and piezoelectric properties for piezoelectric composites

Tensile test specimens were printed with different pretreatment processes and tested using a universal testing machine (ESM303, MARK-10, USA) following Type 5 of the American Society for Testing Material (ASTM) D638 standard. Young's modulus was evaluated from a tensile experiment at a 12.1 mm/min tensile rate for PZT–polymer composites.

The piezoelectric coefficient *d*_33_ was evaluated after the poling process through a quasi-static piezoelectric analyzer (model ZJ-3A, Institute of Acoustics, China) at 110 Hz (frequency) and 0.25 N (force).

### Internal quality analysis of 3D-printed composites

Complex structures with different parameters were printed, and a detailed quality analysis was conducted under optical microscope. We sectioned the printed samples along the *z* axis (printing direction) to assess internal printing quality and inspect the cross-sections. Expected, broadened areas and layer bonding were identified to evaluate the printability. Also, the diameters of the designed and printed rods were measured and compared to evaluate the accuracy.

## Data Availability

The data that support the plots within this paper and other finding of this study are available from the corresponding author upon reasonable request.
